# Peritoneal keratin granuloma associated with endometrioid adenocarcinoma of the uterine corpus

**DOI:** 10.1186/1746-1596-6-104

**Published:** 2011-10-28

**Authors:** Keiichiro Uehara, Masanori Yasuda, Takaya Ichimura, Hiroshi Yamaguchi, Koji Nagata, Hidekazu Kayano, Atsushi Sasaki, Shin-ichi Murata, Michio Shimizu

**Affiliations:** 1 Department of Pathology, Saitama Medical University, International Medical Center, Saitama, Japan; 2 Department of Pathology, Saitama Medical University, Saitama, Japan

**Keywords:** endometrioid adenocarcinoma, peritoneal keratin granuloma, squamous differentiation, uterine corpus

## Abstract

We present a 69-year-old woman with a chief complaint of postmenopausal bleeding. She was diagnosed as having an endometrioid adenocarcinoma by biopsy, and underwent a total abdominal hysterectomy. At the time of surgery, granulation tissue-like nodules were found on the peritoneal serosa of the uterus. In the intraoperative cytology of peritoneal washing, atypical cells were noted. The intraoperative frozen section of the peritoneal nodule revealed granulation tissue with proliferating mesothelial cells. Microscopic examination of the permanent section showed keratin granulomas without viable adenocarcinoma cells on the serosal surface of the ovaries, fallopian tubes and broad ligaments. Postoperative chemotherapy was administered. She has been alive with no evidence of recurrence for 6 months postoperatively. It should be noted that the prognosis of cases in peritoneal keratin granuloma without viable cancer cells is favorable, and that the histological examination is essential for its diagnosis.

## Background

Peritoneal keratin granuloma has been described in cases of endometrioid adenocarcinoma with squamous differentiation of the uterus, ovary and atypical polypoid adenomyoma, although the number is limited [1-9]. These cases may resemble tumor implants grossly and microscopically, and most cases have been described as keratin granulomas without viable tumor cells. Keratin granulomas without viable cancer cells do not show any significant prognostic influence, although the number of cases is limited and the follow-up period is short [2]. In only rare cases, viable adenocarcinomatous cells are found in keratin granulomas [2,6]; however, the treatment in such cases is not established. Here, we report a case of peritoneal keratin granuloma with endometrioid adenocarcinoma of the uterine corpus and review the literature.

## Case presentation

**Figure 1:**
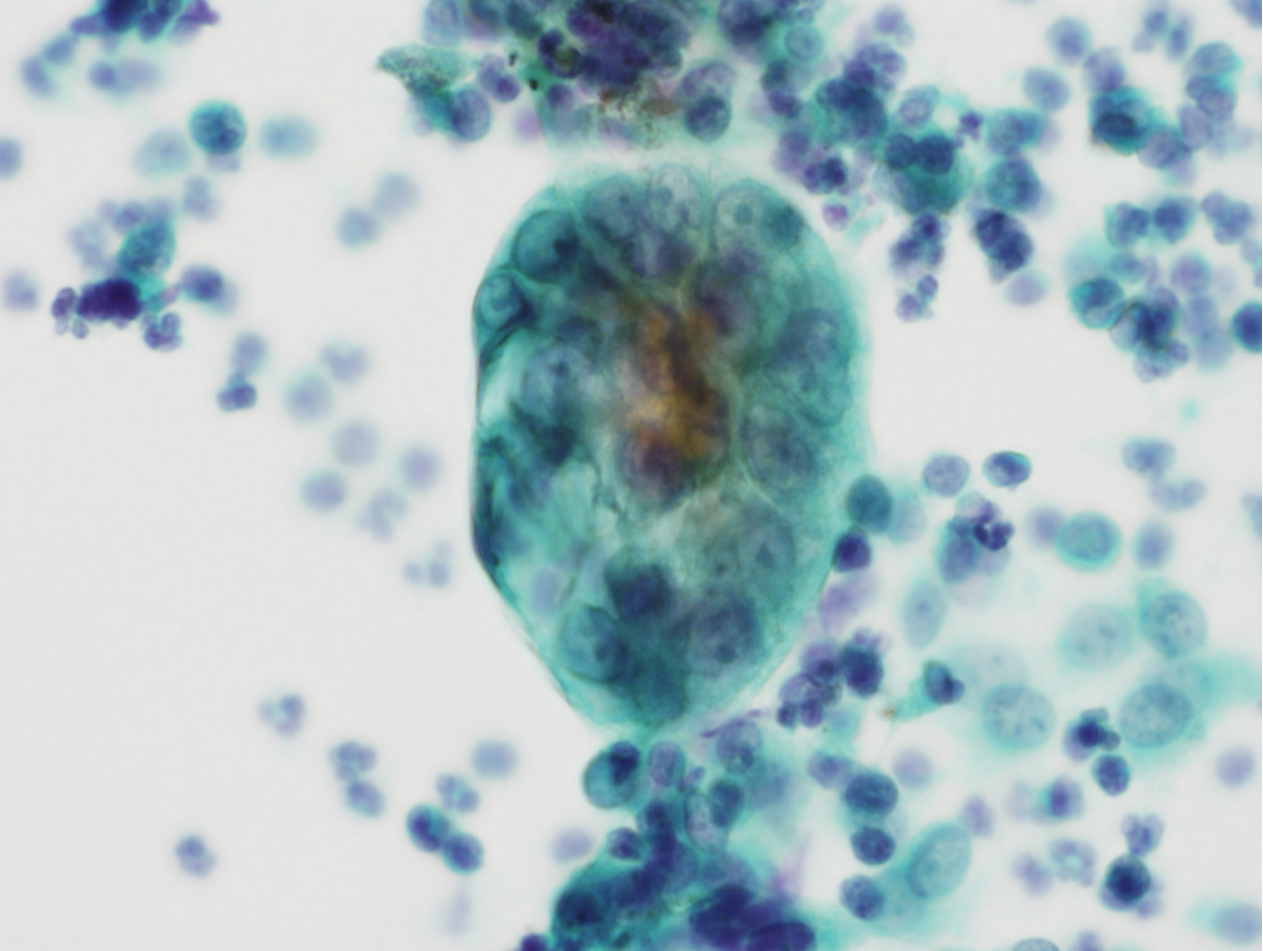
**Intraoperative peritoneal washing cytology shows atypical cells with high N/C ratio and conspicuous nucleoli**. It is difficult to distinguish between reactive mesothelial cells and adenocarcinomatous cells.

A 69-year-old Japanese woman with no previous medical history presented with postmenopausal bleeding. Her first period started at the age of 16, and stopped at the age of 50. She was gravida 2, para 2 (both of them were vaginal deliveries). Cytology and biopsy of the endometrium were obtained, which revealed endometrioid adenocarcinoma. Laboratory data showed no abnormal findings except for the elevation of CA125 (138.2 U/mL, normal < 35.0 U/mL). Total abdominal hysterectomy, bilateral salpingo-oophorectomy, lymph nodes dissection as well as the biopsy of the greater omentum were performed. Atypical cells were noted in the intraoperative cytology of the peritoneal washing (Figure 1). In addition, peritoneal nodules suspicious for dissemination were found. However, it turned out to be granulation tissue with proliferating mesothelial cells in the intraoperative frozen section as well as its permanent section.

Grossly, the uterine corpus showed a papillary mass measuring 8 cm in greatest diameter. Bilateral ovaries, fallopian tubes and broad ligaments showed small grayish nodules measuring about 2 mm in diameter.

**Figure 2:**
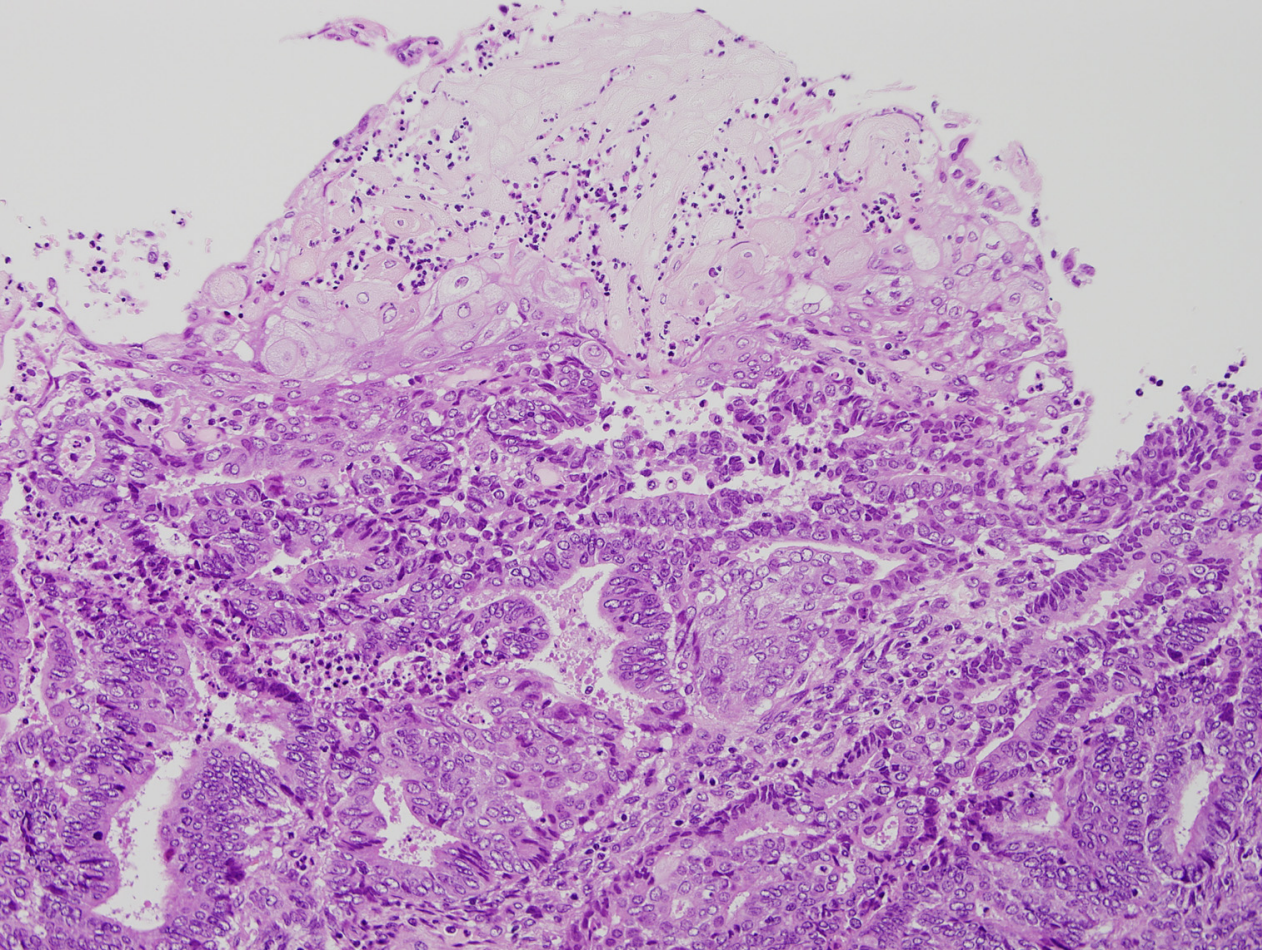
**Well differentiated endometrioid adenocarcinoma showing squamous metaplasia**. Virtual Slide: http://diagnosticpathology.slidepath.com/dih/webViewer.php?snapshotId=1313178756

Histologically, the tumor of the uterine corpus showed diffusely proliferating atypical endometrial glands. Squamous differentiation was also noted (Figure 2). There was no solid growth, and a diagnosis of endometrioid adenocarcinoma (Grade 1) was made. The tumor invaded beyond a half of the myometrium and extended to the uterine cervix (FIGO IIb). Lymph node metastasis was not found.

**Figure 3:**
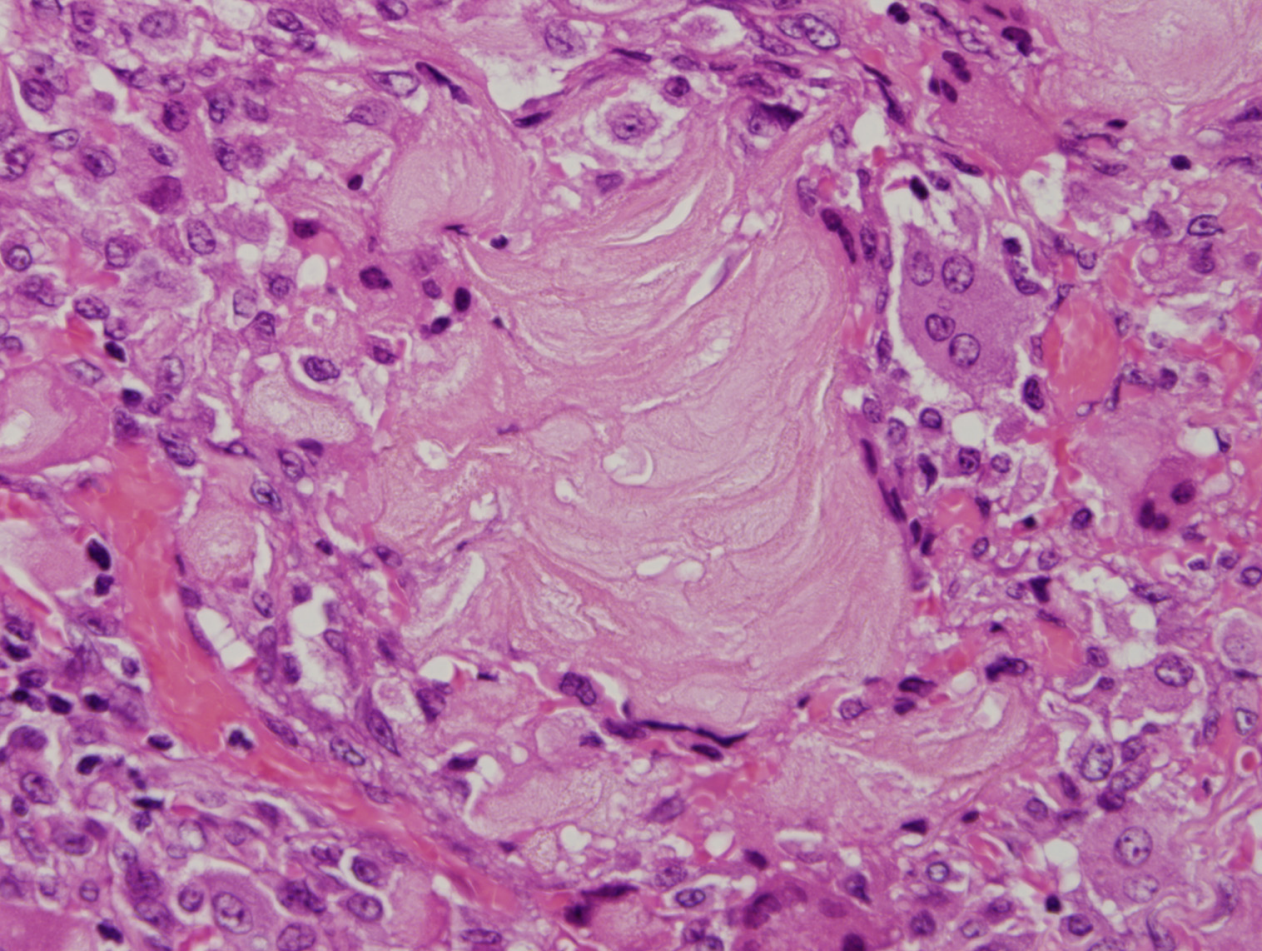
**Keratin granuloma is composed of eosinophilic laminated keratin surrounded by multinucleated giant cells and chronic inflammatory cells**.

**Figure 4:**
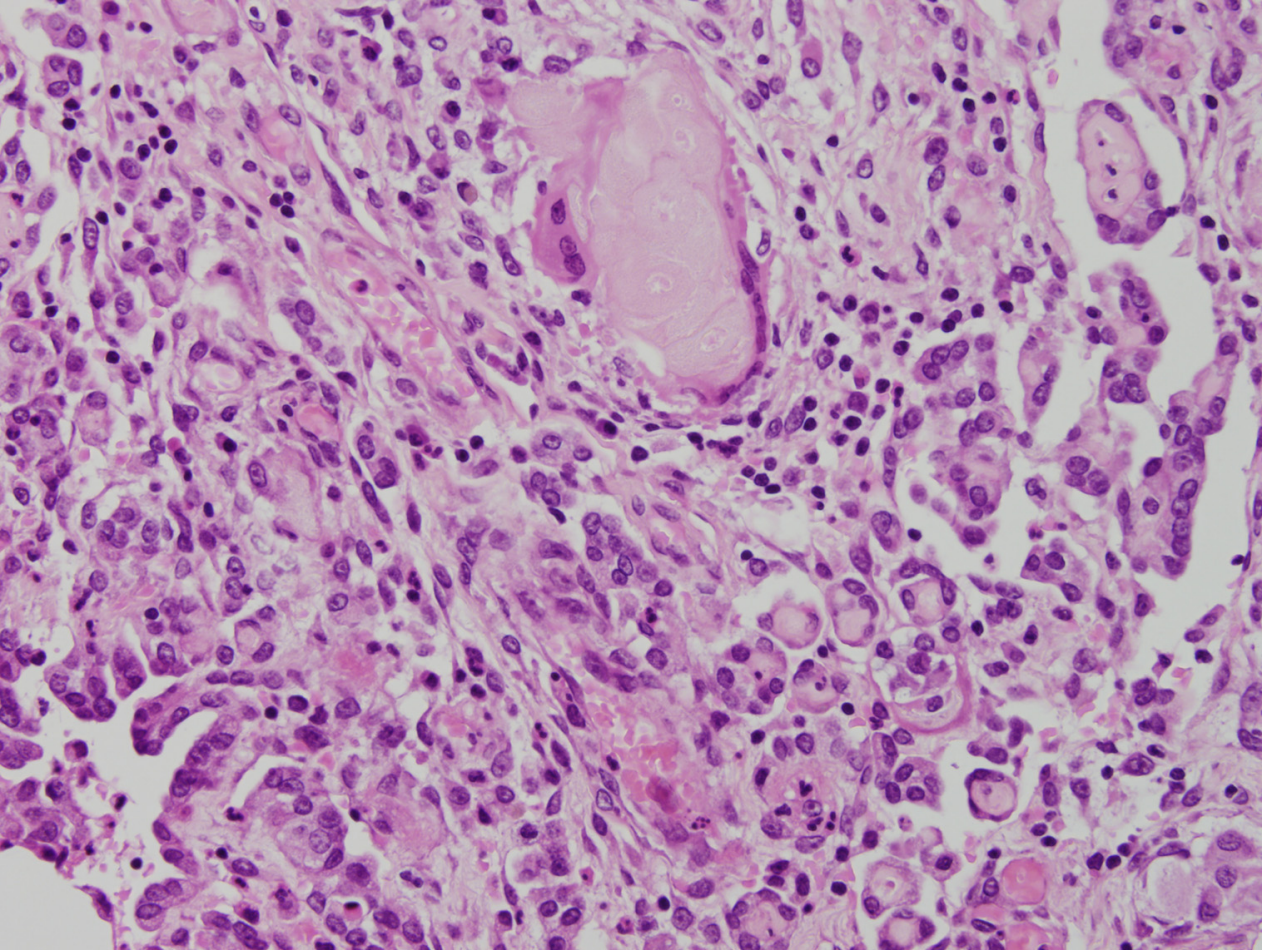
**Reactive mesothelial cells close to keratin granulomas reveal papillary and gland-like structures**.

**Figure 5:**
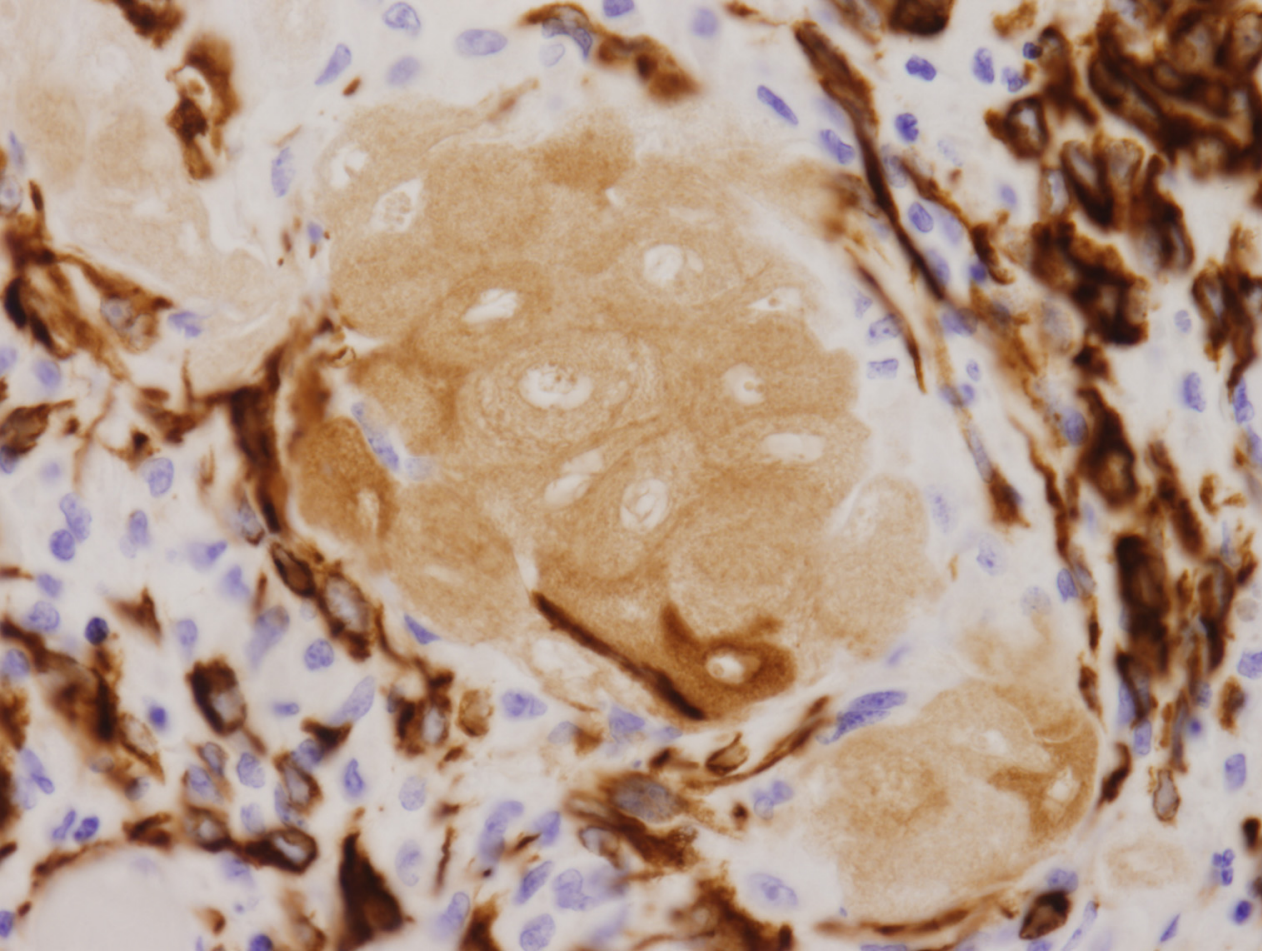
**Immunohistochemically, ghost squamous cells and reactive mesothelial cells are positive for AE1/AE3 in keratin granuloma**.

**Figure 6:**
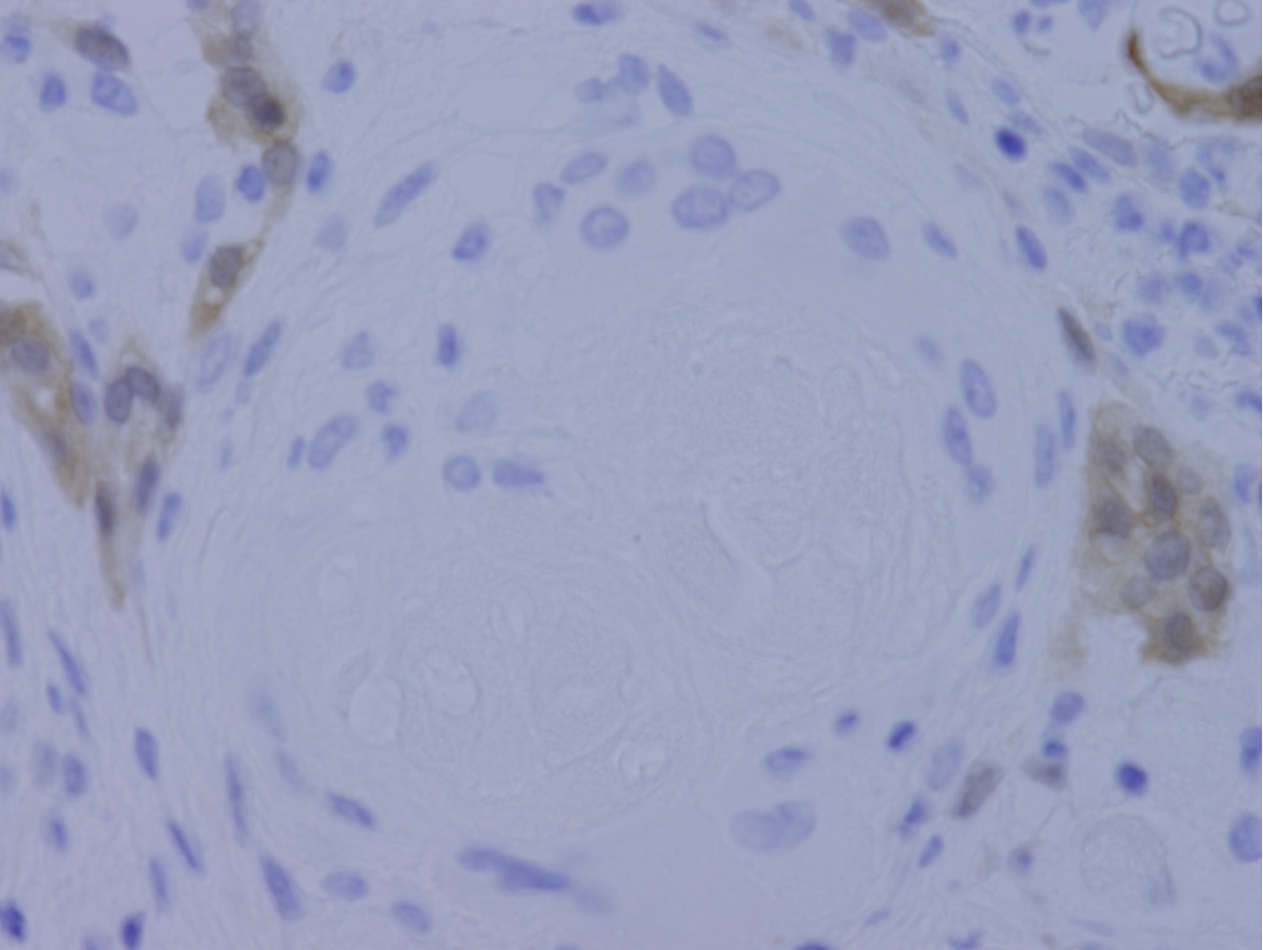
**Immunohistochemically, only reactive mesothelial cells near keratin granuloma for calretinin**.

Granulomas were observed on the serosal surface of the bilateral ovaries, fallopian tubes and broad ligaments, and contained eosinophilic laminated keratin deposits with ghost squamous cells. They were surrounded by epithelioid cells, foreign body type giant cells, neutrophils, lymphocytes and plasma cells (Figure 3). Reactive mesothelial cells with papillary and gland-like structures were also noted (Figure 4). Immunohistochemical stains were performed on the representative section of peritoneal keratin granulomas. The following primary antibodies were used: AE1/AE3 (dilution, 1:50; Dako, Glostrup, Denmark), calretinin (dilution, 1:100; Novocastra, Newcastle, UK), Ber-EP4 (dilution, 1:50; Dako), CAM 5.2 (dilution, prediluted; Becton Dickinson, San Jose, CA), CEA (dilution, 1:50; Dako) and D2-40 (dilution, 1:50; Dako). The ghost squamous cells were positive for only AE1/AE3 (Figure 5). Reactive mesothelial cells were stained with AE1/AE3 (Figure 5), calretinin (Figure 6), CAM 5.2 and D2-40. These reactive mesothelial cells showed higher MIB-1 (dilution, 1:50; Dako) labeling index (10-20%) than surrounding normal mesothelial cells. Ber-EP4 and CEA positive cells were not found within the granulomas. Ziehl-Neelsen and Grocott's methenamine silver stains did not reveal any acid-fast bacilli and fungi.

Postoperative chemotherapy was administered, and she has been alive with no evidence of recurrence for 6 months postoperatively.

## Discussion

Granulomatous inflammation of the peritoneum has been described in cases of nonneoplastic conditions, such as fungal or bacterial infections, ruptured dermoid cyst, previous surgical procedure (talc and starch particles) and diagnostic tests (barium and mineral oil) [1]. In 1961, Montes et al. reported a case of well differentiated adenocarcinoma of the uterine corpus with foreign body keratin granulomas, and named it "cholesteatomatous endometriosis" [3]. In 1978, Chen et al. reported five cases of uterine adenoacanthoma with peritoneal foreign body granulomas to keratin [1]. After that, two additional cases associated with an adenosquamous carcinoma and adenoacanthoma of the uterus were reported in 1984 [5] and 1989, respectively [6].

Histologically, keratin granulomas are composed of eosinophilic laminated keratin surrounded by multinucleated giant cells, histiocytes, lymphocytes and plasma cells. Keratin is associated with ghost squamous cells in which the nuclei have been lost. These keratin materials are speculated to be refluxed from the uterine tumor through the fallopian tubes toward the peritoneum because keratin clumps are often found within the lumen of the fallopian tubes. In addition, many peritoneal keratin granulomas are found around the tubal fimbriae [6].

It is important to recognize keratin granulomas because they resemble metastatic carcinoma or other granulomatous lesion macroscopically. Microscopically, keratin granulomas may be confused with dissemination, since reactive mesothelial cells near the keratin granulomas may proliferate to form papillary or glandular structures [8]. In peritoneal washing cytology, it may be difficult to distinguish between reactive mesothelial cells and tumor cells. In our case, atypical cells of peritoneal washing were subjected to immunohistochemical stain later. Although they were negative for calretinin and CEA, they were most likely reactive mesothelial cells than adenocarcinoma cells.

Peritoneal keratin granulomas have been reported in cases of endometrioid adenocarcinoma with squamous differentiation of the uterine corpus, ovary, and atypical polypoid adenomyoma [1-9]. To our knowledge, only 24 cases of keratin granuloma with endometrioid adenocarcinoma of the uterine corpus have been reported in the English literature (see Additional File 1). Kim et al. reported four cases with synchronous carcinomas of the endometrium and the ovary; however, we excluded these cases because it is uncertain whether the keratin came from the uterus or the ovary [2].

In general, the most common location of granulomas in gynecologic specimens is the surface of the ovary, followed by the serosal surface of the fallopian tube. Postoperative treatments including radiation or chemotherapy were performed in 6 of the cases, and twelve cases were treated with only surgery. One case was treated with surgery and intraperitoneal administration of carboplatin. Clinical follow-up is available for 19 cases. Among them, only one case treated with surgery alone showed a recurrence after a 3-years follow-up, but the patient has been free of the disease for further 10 years afterwards [1]. None of the cases died of the disease.

In three cases with endometrioid adenocarcinoma, viable tumor cells were observed in keratin granulomas [2,6]. These lesions should be regarded as conventional metastatic foci [1]; however, the prognostic significance has not been confirmed because of the short follow-up period. On the other hand, peritoneal keratin granulomas without viable tumor cells do not influence the staging or the prognosis of the primary carcinoma. Therefore, they should not be regarded as an indicator of metastatic spread [1-4]. Only one case reported that adenocarcinomatous cells were detected by careful pathologic examination in an ovarian endometrioid adenocarcinoma [7], which suggests that extensive samplings are essential in the evaluation of these cases.

## Conclusions

Keratin granulomas with endometrioid adenocarcinoma of the uterine corpus resemble a dissemination of tumor cells macro- and microscopically. Keratin granulomas without tumor cells have no significant influences for the prognosis, although the number of these cases and the lengths of the follow-up period are limited. Further studies are needed to establish the significance of keratin granuloma with adenocarcinomatous cells.

## Consent

Written informed consent was obtained from the patient for publication of this Case Report and any accompanying images. A copy of the written consent is available for review by the Editor-in-Chief of this journal.

## Competing interests

The authors declare that they have no competing interests.

## Authors' contributions

KU drafted the manuscript. MS participated in the design of study. MY, IT, HY, KN, HK, AS and SM helped to draft the manuscript. All authors read and approved the final manuscript.

## Supplementary Material

Additional file 1 **Clinicopathological features of reported cases of keratin granuloma with endometrioid adenocarcinoma of the uterine corpus**. The table shows reported 24 cases of keratin granuloma with endometrioid adenocarcinoma of the uterine corpus. Click here for file
